# Supplementary material for: Standardized Extract from Wastes of Edible Flowers and Snail Mucus Ameliorate Ultraviolet B-Induced Damage in Keratinocytes
Source: Int J Mol Sci. 2023 Jun 15;24(12):10185. doi: 10.3390/ijms241210185 (PMC10298975; doi:10.3390/ijms241210185)
Supplement: Supplementary file 1 [file ijms-24-10185-s001.zip › ijms-2428021-supplementary.pdf]

Communication

SUPPLEMENTARY MATERIALS

Standardized Extract from Wastes of Edible Flowers and Snail Mucus Ameliorate Ultraviolet B-Induced Damage in Keratinocytes

Luca Vanella [1,2,*,†], Valeria Consoli [1,2,†], Ilaria Burò [1], Maria Gulisano [1], Manuela Stefania Giglio [1], Ludovica Maugeri [1], Salvatore Petralia [1], Angela Castellano [3] and Valeria Sorrenti [1,2]

[1] Department of Drug and Health Sciences, University of Catania, 95125 Catania, Italy; valeria_consoli@yahoo.it (V.C.); ilariaburo95@gmail.com (I.B.); maria.gulisano@hotmail.it (M.G.); manuela1189glg@gmail.com (M.S.G.); maugeriludovica@gmail.com (L.M.); salvatore.petralia@unict.it (S.P.); sorrenti@unict.it (V.S.)

[2] CERNUT–Research Centre for Nutraceuticals and Health Products, University of Catania, 95125 Catania, Italy

[3] Mediterranean Nutraceutical Extracts (Medinutrex), Via Vincenzo Giuffrida 202, 95128 Catania, Italy; info@medinutrex.com

* Correspondence: lvanella@unict.it

† These authors contributed equally to this work.

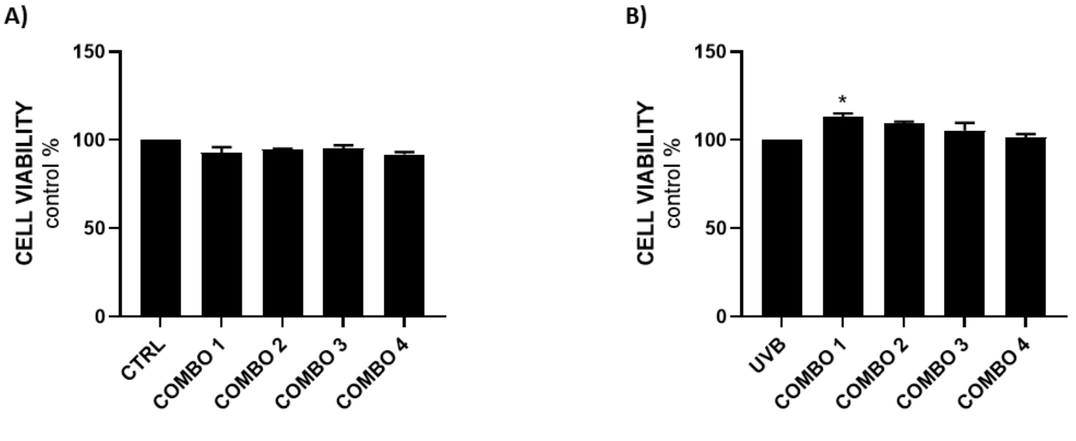

Figure S1. Assessment of cell viability after combination treatment (COMBO 1 : SEM 5 ug/ml + EFE 0.25 mg/ml ; COMBO 2: SEM 5 ug/ml + EFE 0.5 mg/ml; COMBO 3: SEM 10 ug/ml + EFE 0.25 mg/ml; COMBO 4: SEM 10 ug/ml + EFE 0.5 mg/ml) in cells exposed (**B**) or not (**A**) to UVB radiation (100 sec). * $p < 0.05$.

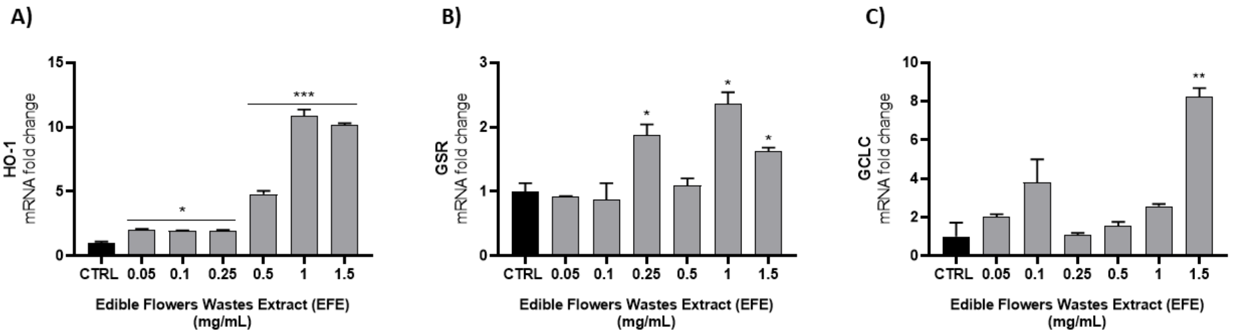

Figure S2. (A-C) Evaluation of EFE's effect on antioxidant genes' expression following 24h treatment. Results are expressed as mean ± SEM. (* $p < 0.05$; ** $p < 0.005$; *** $p < 0.0005$ vs CTRL)

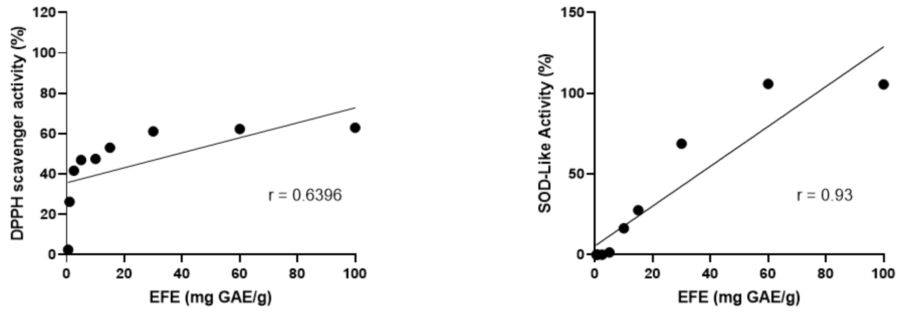

Figure S3. Pearson correlation scatter plot of relationship between antioxidant activity and total polyphenols content of EFE.
